# Overexpression of *Calcineurin* B-like Interacting Protein Kinase 31 Promotes Lodging and Sheath Blight Resistance in Rice

**DOI:** 10.3390/plants13101306

**Published:** 2024-05-09

**Authors:** Jingsheng Chen, Siting Wang, Shiqi Jiang, Tian Gan, Xin Luo, Rujie Shi, Yuanhu Xuan, Guosheng Xiao, Huan Chen

**Affiliations:** 1 College of Biology and Food Engineering, Chongqing Three Gorges University, Wanzhou 404100, China; jingshengchen@sanxiau.edu.cn (J.C.); xiaojiangshiqi@126.com (S.J.); gantian0118@163.com (T.G.); xin_luo0316@163.com (X.L.); yusrj@163.com (R.S.); 2 College of Plant Protection, Shenyang Agricultural University, Shenyang 110866, China; 2021200147@stu.syau.edu.cn; 3 State Key Laboratory of Elemento-Organic Chemistry, Nankai University, Tianjin 300071, China; 9920230120@nankai.edu.cn; 4 Department of Plant Protection, National Pesticide Engineering Research Center (Tianjin), Nankai University, Tianjin 300071, China; 5 Key Laboratory of Saline-Alkali Vegetation Ecology Restoration, Ministry of Education, Northeast Forestry University, Harbin 150040, China

**Keywords:** *CIPK31*, sheath blight, lodging, resistance, rice

## Abstract

A breakthrough “Green Revolution” in rice enhanced lodging resistance by using gibberellin-deficient semi-dwarf varieties. However, the gibberellic acid (GA) signaling regulation on rice disease resistance remains unclear. The resistance test showed that a positive GA signaling regulator *DWARF1* mutant *d1* was more susceptible while a negative GA signaling regulator *Slender rice 1* (*SLR1*) mutant was less susceptible to sheath blight (ShB), one of the major rice diseases, suggesting that GA signaling positively regulates ShB resistance. To isolate the regulator, which simultaneously regulates rice lodging and ShB resistance, SLR1 interactors were isolated. Yeast two-hybrid (Y2H), bimolecular fluorescence complementation (BiFC), and Co-IP assay results indicate that SLR1 interacts with Calcineurin B-like-interacting protein kinase 31 (CIPK31). *cipk31* mutants exhibited normal plant height, but *CIPK31 OXs* showed semi-dwarfism. In addition, the SLR1 level was much higher in *CIPK31 OXs* than in the wild-type, suggesting that *CIPK31 OX* might accumulate SLR1 to inhibit GA signaling and thus regulate its semi-dwarfism. Recently, we demonstrated that CIPK31 interacts and inhibits Catalase C (CatC) to accumulate ROS, which promotes rice disease resistance. Interestingly, CIPK31 interacts with Vascular Plant One Zinc Finger 2 (VOZ2) in the nucleus, and expression of CIPK31 accumulated VOZ2. Inoculation of *Rhizoctonia solani* AG1-IA revealed that the *voz2* mutant was more susceptible to ShB. Thus, these data prove that CIPK31 promotes lodging and ShB resistance by regulating GA signaling and VOZ2 in rice. This study provides a valuable reference for rice ShB-resistant breeding.

## 1. Introduction

Enhancement of yield and immunity are two vital factors of plant breeding. Nevertheless, the signaling pathways that control these processes are often antagonistically controlled, which poses challenges for breeding strategies targeting increased yield and disease resistance in rice [1]. Plants rely on their innate immune system to detect and defend against threats, yet this immunity is closely linked to plant growth and development.

Previous studies have reported that plant defense and immunity are regulated in opposing ways. In *Arabidopsis*, XLGs are functional Gα (Gα subunits) proteins that are localized in the nucleus and can interact with Gβγ dimers to form heterotrimers [2,3,4,5]. These heterotrimers play a crucial role in various aspects of plant growth, including cell division, maintenance of meristems, root morphogenesis, seed development and germination, nitrogen assimilation, responses to ABA and auxin as well as abiotic stress [2,6,7,8,9,10,11,12,13,14,15]. XLG proteins are also essential for pathogen protection [3,4,16]. OsXLG1 positively regulates the immune response of rice against the bacterial blight pathogen *Xanthomonas oryzae* pv. *oryzae* (*Xoo*) while simultaneously negatively affecting plant height and spike length in rice through a chitin-induced defense response [17]. Phospholipid metabolism is implicated in disease resistance in rice. *RESISTANCE TO BLAST1* (*RBL1*), encoding CDP-DAG synthase, is vital for phospholipid production and regulates phosphatidylinositol levels, influencing programmed cell death (PCD) and contributing significantly to plant immunity [18]. A 29 bp deletion in *RBL1* results in reduced levels of phosphatidylinositol and its derivative phosphatidylinositol 4,5-bisphosphate (Ptdlns(4,5)P2), a factor contributing to disease susceptibility [19]. RBL1-mediated signaling enhances broad-spectrum disease resistance in rice, while it also contributes to a significant reduction in yield by approximately 20-fold [20]. ROD1, also known as RESISTANCE OF RICE TO DISEASES1, is a C2 domain Ca^2+^ sensor that interacts with catalase, the CatB, and enhances the scavenging of reactive oxygen species (ROS). ROD1-CatB is active in moderating ROS bursts in response to pathogen infestation. Moreover, two E3 ubiquitin ligases, RIP1 and APIP6, degrade ROD1 and maintain ROS production, thereby enhancing broad-spectrum disease resistance. However, this mechanism delays the development of spike meristematic tissues, leading to lower yields [21].

Achieving a balance between yield and resistance during crop production is a persistent issue [22]. PRE-IBH1-HBI1 module sacrifices immune regulation to mediate plant growth regulation through responding to hormone and environmental signals [23]. In the salicylic acid signaling pathway, *WRKY45* is activated by multiple transcription factors to mediate resistance to various pathogens, including *Magnaporthe oryzae* [24]. The ubiquitin-conjugating enzyme OsUBC26 plays a role in the degradation of the *M. oryzae* effector protein AvrPiz-t by participating in proteasome assembly [25]. However, a balance between disease resistance and yield increase can be achieved through the regulation of individual genes. OsUBC45, a protein localized in the endoplasmic reticulum (ER), can target OsPIP2;1 for degradation, which raises pathogen-associated molecular pattern-triggered immunity (PTI) and improves rice resistance to *M. oryzae* and bacterial blight. This leads to an increase in yield of more than 10% while resisting these diseases [26]. Additionally, increasing the expression level of *Ideal Plant Architecture1* (*IPA1*) to confer blast resistance can increase rice yield [27,28]. Overexpression of *Lose Plant Architecture 1* (*LPA1*) promotes rice resistance to ShB and planting density by activation of *PIN-FORMED 1a* (*PIN1a*) [29]. Dense and Erect Panicle 1 (DEP1) interacts and inhibits LPA1 DNA-binding activity to negatively regulate rice resistance to ShB and planting density [30]. Ammonium transporter 1 (AMT1) promotes rice resistance to ShB and yield production by activation of N-metabolism and ethylene signaling [31]. Tissue-specific activation of *DOF11* promotes rice yield and ShB resistance [32]. *OsUPM1* is known to encode a proteasome maturation factor that enhances the abundance and activity of the 26S proteasome biosynthesis, which in turn promotes the degradation of peroxidase APX8 and catalase CatB in response to pathogen invasion. This results in the accumulation of H_2_O_2_ and improved resistance of rice against the ShB, blast and bacterial blight pathogen *Xoo*, without any penalization on grain yield [33]. OsVQ25, a protein containing the valine-glutamine (VQ) motif, can interact with the U-Box E3 ligase OsPUB73 and the transcription factor OsWRKY53. OsPUB73 facilitates OsVQ25 degradation via the 26S proteasome pathway while reducing *VQ25* enhances resistance to blast and pathogen *Xoo*. Additionally, OsWRKY53 regulates downstream defense and BR signaling pathway genes, which are upregulated and contribute to maintaining rice yield and development [34]. The agricultural green revolution of the 1960s enhanced cereal crop yields by cultivation of semi-dwarf crop varieties e.g., *sd1* mutant in rice [35,36]. However, the disease resistance of semi-dwarf cultivars has not been much studied.

This study investigated the role of GA signaling in rice resistance. We screened SLR1-interacting proteins and examined the function of the SLR1-interactor CIPK31 in plant height and rice resistance. *CIPK31* overexpression led to semi-dwarfism due to SLR1 accumulation, while enhancing rice resistance by activating VOZ2. These findings suggest that *CIPK31* could serve as a valuable gene source for improving rice lodging and ShB resistance.

## 2. Results

### 2.1. GA Signaling Positively Regulates ShB Resistance

**Figure 1:**
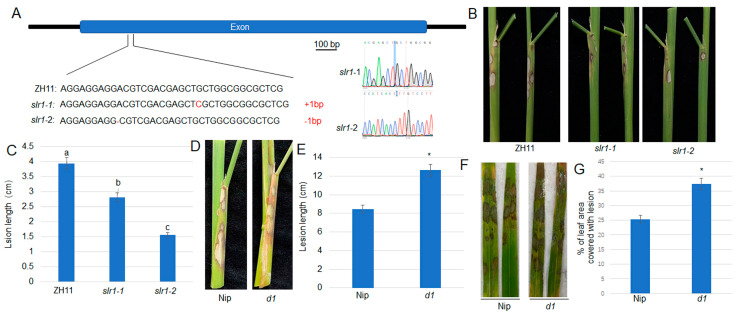
GA signaling positively regulates rice ShB resistance. (**A**) CRISPR/Cas9-induced genome editing in *SLR1* mutants (*slr1-1* and *slr1-2*). The peak of sequencing results within edited region were shown. (**B**) The sheaths of ZH11 and *slr1* mutants (*-1*, *-2*) inoculated with *R. solani* AG1-IA. (**C**) The length of lesions on the sheaths shown in (**B**). (**D**) The sheaths of Nipponbare (Nip) and *d1* inoculated with *R. solani* AG1-IA. (**E**) The length of lesions on the sheaths shown in (**D**). (**F**) Nipponbare and *d1* leaves inoculated with *R. solani* AG1-IA. (**G**) The percentage of lesions on leaves shown in (**F**). Different lowercase letters and asterisks represent statistically significant differences; *p* < 0.05.

The yield and immunity were the important factors in rice production. Rice green revolution was mainly achieved by the application of GA biosynthetic gene mutants *sd1* which greatly improved crop yield [36,37]; however, the GA signaling response to ShB, a major rice disease is not much understood. To further investigate GA signaling in rice resistance, the CRISPR/Cas9-mediated *SLR1* genome editing mutants were generated. SLR1 is degraded by the activation of GA signaling, which is a negative regulator of GA signaling [38]. GA regulates cell elongation, and the *slr1* mutant exhibited higher plant by activating GA signaling [39]. The sequencing results showed that 1 bp insertion and 1 bp deletion were observed in *slr1-1* and *slr1-2*, respectively (Figure 1A). Inoculation of *R. solani* AG1-IA revealed that *slr1* mutants are less susceptible to ShB (Figure 1B,C). Since SLR1 is a negative regulator of GA signaling, the function of *Dwarf1* (*D1*), a positive regulator of GA signaling in ShB defense was analyzed. *d1* mutant significantly shorter than wild-type control, and in which GA signaling transduction was blocked [40]. Inoculation of *R. solani* AG1-IA indicated that the *d1* mutant was more susceptible to ShB compared to wild-type plants Nipponbare (Nip) (Figure 1D–G).

### 2.2. CIPK31 Interacts with SLR1 to Modulate Plant Height

**Figure 2:**
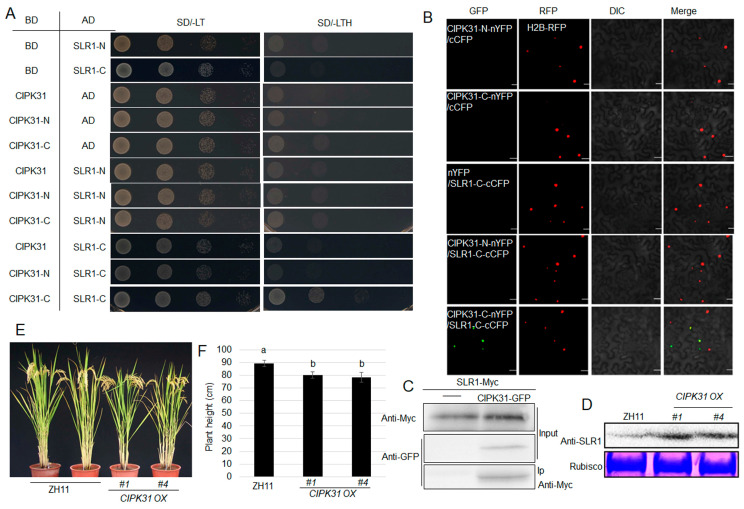
CIPK31 interacts and stabilizes SLR1. (**A**) Yeast two-hybrid assays for interaction between N- or C-terminal of CIPK31 and N- or C-terminal of SLR1. (**B**) BiFC assay for interaction between CIPK31 C-terminal and SLR1 C-terminal region in tobacco leaves and YFP reconstruction. Bar = 20 μm. (**C**) Co-IP assay for interaction between CIPK31-GFP and SLR1-Myc in tobacco. CIPK31-GFP + SLR1-Myc or SLR1-Myc alone was expressed in tobacco leaves, and the total protein was immunoprecipitated using an anti-Myc antibody. The input and immunoprecipitated proteins were detected using anti-GFP or anti-Myc antibodies. (**D**) The SLR1 protein level detected using an anti-SLR1 antibody in ZH11 and *CIPK31 OXs (#1*, *#4*). Coomassie brilliant blue (CBB) staining of Rubisco was used as the loading control. (**E**) Three-month-old ZH11 and *CIPK31 OXs* (*#1*, *#4*). (**F**) The height of plants shown in (**E**). Different lowercase letters indicate significant differences at *p* < 0.05.

Examination of *slr1* and *d1* mutants identified that GA signaling positively regulates rice ShB resistance. It suggests that plant height and resistance are positively correlated in GA signaling. To identify the regulators promoting both semi-dwarfism and resistance, the yeast two-hybrid screening was performed using SLR1 as a bait. Among the SLR1 interactors, CIPK31 interacts with SLR1. Further Y2H and BiFC assays showed that the C-terminal region of CIPK31 interacts with the C-terminal of SLR1 in the nucleus (Figure 2A,B). A Co-IP assay was performed by expressing SLR1-Myc alone or coexpressing CIPK31-GFP with SLR1-Myc in tobacco leaves. Anti-Myc antiserum was used for immunoprecipitation, and anti-GFP and anti-Myc antibodies were used to detect protein. Western blot analysis showed an interaction between CIPK31 and SLR1 (Figure 2C). The *CIPK31 OX* plants exhibited a semi-dwarf morphology compared to wild-type plants (Figure 2E,F), while *cipk31* and the wild type had similar plant heights. Moreover, the *CIPK31 OXs* accumulated higher SLR1 protein levels than the wild-type plants (Figure 2D).

### 2.3. CIPK31 Interacts with and Stabilizes VOZ2

**Figure 3:**
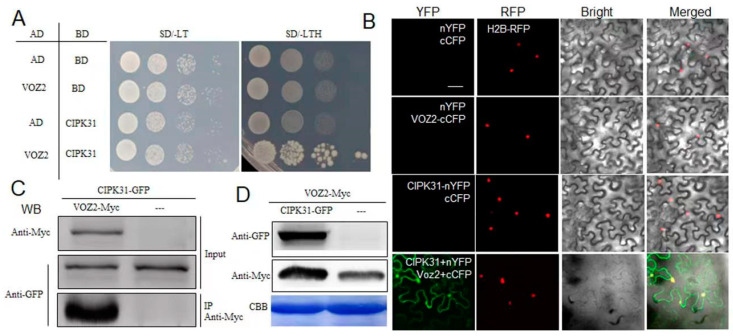
CIPK31 interacts with VOZ2. (**A**) Yeast two-hybrid assay for CIPK31 interactions with VOZ2. (**B**) BiFC assay for CIPK31 and VOZ2 interaction. nYFP + cCFP, nYFP + VOZ2-cCFP, CIPK31-nYFP + cCFP, or CIPK31-nYFP + VOZ2-cCFP were coexpressed in tobacco leaves, and YFP reconstruction was examined. Bar = 20 μm. H2B-RFP was colocalized as the nuclear marker. (**C**) Co-IP assay for interaction between CIPK31-GFP and VOZ2-Myc in tobacco. CIPK31-GFP+VOZ2-Myc or CIPK31-GFP alone was expressed in tobacco leaves, and the total protein was immunoprecipitated using an anti-GFP antibody. The input and immunoprecipitated proteins were detected using anti-GFP or anti-Myc antibodies. (**D**) VOZ2-Myc or CIK31-GFP and VOZ2-Myc were expressed in the tobacco leaves. The Western blot analysis was performed to detect CIPK31-GFP and VOZ2-Myc using the anti-GFP and anti-Myc antibodies, respectively. Coomassie brilliant blue (CBB) staining of Rubisco was used as the loading control.

We previously demonstrated that CIPK31 interacts and inhibits Catalase C to accumulate ROS by which CIPK31 promotes rice disease resistance [41]. Together with these results, *CIPK31* overexpression plants promote both semi-dwarfism and ShB resistance. However, *CIPK31* overexpression accumulated the SLR1 protein level thus inhibited GA signaling, suggesting that CIPK31’s promotion of ShB resistance is not associated with SLR1. To dissect CIPK31 regulation of rice ShB resistance, the CIPK31 interacting proteins were dissected. Among the CIPK31 interactors, interestingly, VOZ2 also interacted with CIPK31 (Figure 3A). BiFC assays showed that CIPK31 and VOZ2 interact in the cytosol and nucleus in tobacco leaves (Figure 3B). A Co-IP assay was performed by expressing CIPK31-GFP alone or coexpressing CIPK31-GFP with VOZ2-Myc in tobacco leaves. Anti-GFP antiserum was used for immunoprecipitation, and anti-GFP and anti-Myc antibodies were used to detect protein. Western blot analysis showed an interaction between CIPK31 and VOZ2 (Figure 3C). To investigate the role of CIPK31 on VOZ2, VOZ2-Myc alone or CIPK31-GFP and VOZ2-Myc were coexpressed in the tobacco leaves. Western blot analysis showed that expression of CIPK31 accumulated more VOZ2 (Figure 3D).

**Figure 4:**
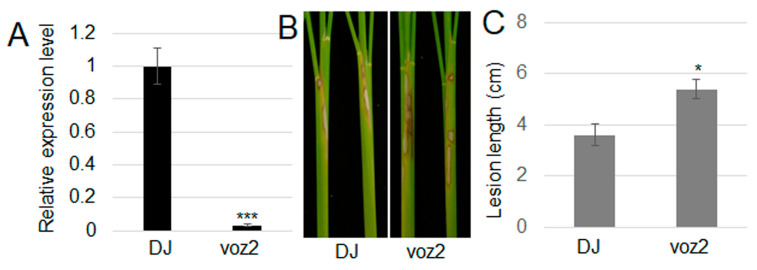
*voz2* mutant was more susceptible to ShB. (**A**) *VOZ2* expression level was examined in Dongjin (DJ) and *voz2* mutant by RT-qPCR. (**B**) The sheaths of DJ and *voz2* mutant inoculated with *R. solani* AG1-IA. (**C**) The length of lesions on the sheaths shown in (**B**). Different asterisks represent statistically significant differences; *p* < 0.05.

Therefore, to test *VOZ2* function in rice resistance against ShB, a *voz2* T-DNA mutant in Dongjin (DJ) background was used [42]. RT-qPCR showed that almost no expression of *VOZ2* was detected in *voz2* mutant compared to wild-type DJ (Figure 4A). Inoculation of *R. solani* AG1-IA revealed that *voz2* mutant plants were more susceptible to ShB than DJ (Figure 4B,C), suggesting that CIPK31 might interact and stabilize VOZ2 to enhance rice resistance to ShB.

## 3. Discussion

Rice yield and immunity are the key factors in breeding; however, the signaling pathways that control yield and immunity are often antagonistically regulated; therefore, there are difficulties in improving both yield and immunity [1]. The agricultural green revolution of the 1960s enhanced cereal crop yields by the cultivation of semi-dwarf crop varieties [35]. Rice green revolution was mainly achieved by planting *sd1* mutant which reduces cellular gibberellin levels [36,37]. Cultivation of *sd1* by reducing plant height significantly increased rice yield and lodging resistance, which made a significant contribution to the development of agriculture. However, how GA signaling regulated rice immunity was not much understood.

ShB as a disease model, we have examined the ShB resistance of mutants of *SLR1* and *DWAF1*, negative and positive GA regulators, respectively. The data indicate that GA signaling positively regulates rice resistance to ShB, suggesting that green revolution by reducing GA biosynthesis could increase yield but reduce resistance to ShB in rice. To isolate the regulators for increasing both resistance to lodging and immunity, *SLR1*, a negative GA signaling gene was used as a bait for yeast two-hybrid screening. Interestingly, CIPK31 interacts with SLR1. Recently, we identified that *CIPK31 OXs* showed a ShB resistance compared to wild-type plants [41]. Interestingly, the CIPK31 C-terminal interacted with SLR1. After GID1, a soluble receptor perceives the GA molecule, which triggers the degradation of SLR1 to activate downstream GA signaling [43]. Studies have shown that CKI phosphorylates SLR1 to negatively regulate GA signaling, suggesting that phosphorylation of SLR1 is crucial for its stability [44]. Western blot analysis indicated that the SLR1 protein level was significantly higher in *CIPK31 OX* plants, implying that CIPK31 might interact with SLR1 to inhibit its degradation. Since SLR1 interacts with the C-terminal of CIPK31, not a kinase domain, the mechanism via which CIPK31 stabilizes SLR1 needs to be further investigated. However, *cipk31* mutants and wild-type plants had similar heights, implying that CIPK31 is not the major regulator of rice height.

Earlier, a mutation at *SD1*, a GA biosynthetic enzyme, resulted in a high-yielding semi-dwarf rice plant called “green evolution” [37]. Semi-dwarfness increases rice resistance to lodging, greatly benefiting breeding. ROD1 interacts with CatB to negatively regulate a broad-spectrum resistance; however, *rod1* showed obvious growth penalties, resulting in reduced yield. Natural *ROD1* alleles enhance resistance without affecting yield [21,45]. These observations implied a balance between growth and defense [1] and these genes’ weaknesses in rice resistance breeding. We found that CIPK31 regulates cellular ROS levels to promote rice resistance [41] and reduce height, and high CIPK31 levels stabilize SLR1. However, different from *rod1* and *catB*, the *catC* mutants and *CIPK31 OXs* maintained normal yield, suggesting that CIPK31 might inhibit CatC at the specific tissues which increase resistance without yield penalty in *CIPK31 OXs* or CIPK31 might partially regulate multiple signaling pathways to modulate rice yield and immunity, which implying a great potential for *CIPK31* in rice resistance breeding.

Previous reports showed that *CIPK31* expression level was positively associated with cellular H_2_O_2_ levels, and further analyses identified that CIPK31 interacts with and inhibits CatC from accumulating cellular ROS [41]. Screening for CIPK31-interacting proteins identified that VOZ2, a transcriptional activator which previously reported to positively regulate rice blast resistance, and the RING-type E3 ligase AVRPIZ-T INTERACTING PROTEIN 10 (APIP10) negatively regulates rice blast resistance by degradation of VOZ2 [42]. Inoculation of *R. solani* AG1-IA also revealed that VOZ2 promoted rice resistance to ShB, suggesting that VOZ2 might regulate a broad-spectrum resistance in rice. A few reports demonstrated the interaction between CIPKs and transcription factors. CIPK11 interacts with and phosphorylates the FIT transcription factor, promoting iron acquisition [46], and CIPK26 phosphorylates ABI5 to regulate ABA signaling in *Arabidopsis* [47]. In the CIPK31 interactors, we also identified VOZ1 [48], suggesting that CIPK31 might interact with VOZs to modulate rice defense. Coexpression of CIPK31 stabilized VOZ2, implying that CIPK31 might inhibit APIP10-dependent VOZ2 degradation to stabilize it. CIPK31 is a protein kinase; therefore, it might interact with and phosphorylate VOZs to modulate their transcriptional regulation. Furthermore, BiFC data showed that CIPK31 interacts with VOZ2 at the cytosol and nucleus. CIPK31 is localized at the cytosol and nucleus [48], and VOZ2 is a transcription factor localized at the nucleus [42]. Further study would be valuable to clarify the detailed regulatory mechanism of CIPK31 on VOZ2 in the cytosol and nucleus. In addition, the interaction domain tests demonstrated that the CIPK31-C terminal interacts with the VOZ2-C terminal. The CIPK31 consists of an N-terminal kinase domain and a C-terminal regulatory domain. These data imply that CIPK31 might not interact with and phosphorylate VOZ2, but CIPK31 might interact with VOZ2 to block APIP10 binding to VOZ2 by which CIPK31 stabilizes VOZ2.

Taken together, GA signaling promotes rice height and ShB resistance; however, the increase in plant height results in the loading. Interestingly, CIPK31 interacts with SLR1, a negative regulator of GA signaling to promote plant semi-dwarfism. In addition, CIPK31 interacts and stabilizes VOZ2 to improve the rice ShB resistance. These data provided an interesting finding that CIPK31 might regulate multiple pathways to control rice plant height and ShB resistance, which provided a useful source for breeding to avoid a “trade-off” between yield and immunity.

## 4. Materials and Methods

### 4.1. Plant Growth and Pathogen Inoculation

The experimental plants are planted between approximately 30°23′50″ N to 31°0′18″ N and 107°52′22″ E to 108°53′52″ E. The wild-type (WT) rice cultivars (*Oryza sativa* L. Japonica cultivars Zhonghua 11, Nipponbare, and Dongjin) and the *slr1*, *d1*, *voz2*, *cipk31*, *CIPK31 OX* lines were used in this study. The *slr1* mutants were constructed using CRISPR/Cas9 genome editing technology in the Zhonghua11 background (Baige Gene Technology, Changzhou, China) (Figure 1A). The preparation of *d1*, *voz2*, *cipk31*, and *CIPK31 OXs* plants was described previously [41,42,49]. The plants were grown in the glass cultivation room of Chongqing Three Gorges University under controlled conditions at 24–30 °C, 70% relative humidity, and 12 h of light. *Nicotiana benthamiana* plants were cultivated in growth rooms at 25 °C under a 16-h light/8-h dark cycle. The *R. solani* isolate AG1-IA was pre-incubated on potato dextrose agar (PDA) at 26 °C for 2–4 days before inoculation. All rice plants were examined for resistance to leaf blight (ShB) at the third leaf stage using the method described [50]. The leaves were generally photographed 48 h after inoculation. The leaf sheath part is usually photographed 10 days after inoculation. Lesion lengths in the leaves or leaf sheaths were calculated after photos were taken. For lesion quantification, ImageJ software (version 1.5.3) was utilized for analysis.

### 4.2. RNA Extraction and Reverse Transcription-Polymerase Chain Reaction (RT-qPCR)

Total RNA was isolated from one-month-old rice leaves using TRIzol reagent (Takara, Dalian, China), and the genomic DNA contamination was removed using the RQ-RNase-free DNase (Promega, Madison, WI, USA). Complementary DNA was synthesized from the extracted RNA using GoScript Reverse Transcription Kit (Promega) according to the manufacturer’s instructions. The gene expression levels were determined using qRT-PCR performed with SYBR-Green (Takara) on a BIO-RADCFX96 real-time PCR system (Bio-Rad, Hercules, CA, USA), normalizing to ubiquitin levels. A minimum of three biological replicates and two technical replicates were used for each analysis. Relative expression levels were calculated using the 2^−ΔΔCt^ method. Primers used for RT-qPCR are listed in the supplemental Table S1.

### 4.3. Protein Extraction and Western Blot Analysis

Tissues collected from one-month-old rice plants were thoroughly ground in liquid nitrogen, and 1 g of each sample was lysed with 200 μL of 2× SDS sample buffer to extract the proteins. Samples were then centrifuged at 12,000 rpm and 4 °C for 10 min, and the supernatant was removed to a fresh tube. The protein content of the supernatant was quantified using Modified Bradford Protein Assay Kit (Sangon Biotech, Shanghai, China). The protein (20 µg) was separated on a 10% SDS-PAGE gel and electrotransferred onto Immobilon-P Transfer Membrane (MILLIPORE JAPAN, Tokyo, Japan) for subsequent Western blot analysis, using the following primary antibodies: anti-SLR1 antibody (1:2000; Abclonal, Wuhan, China), anti-GFP antibody (1:2000; Sigma, Tokyo, Japan), and anti-Myc antibody (1:2000; Sigma). The membranes were subsequently incubated for an hour with anti-mouse or anti-rabbit horseradish peroxidase (HRP)-conjugated secondary antibody (1:2000; Cell Signaling Technology, Danvers, MA, USA), and the signal was detected using an ECL Western Blotting Detection System (GE Healthcare, Piscataway, NJ, USA).

### 4.4. Co-Immunoprecipitation Assay (Co-IP) and Western Blot Analyses

The Myc+CIPK31-GFP and SLR1-Myc + CIPK31-GFP or VOZ2-Myc + CIPK31-GFP and Myc + CIPK31-GFP constructs were coexpressed in *Nicotiana benthamiana* leaves using the Agrobacterium-mediated transient expression. Each interaction pair was mixed in a 5:5:2 ratio with P19, a silencing suppressor, and infiltrated tobacco leaves. After 48 h, 2 g leaf samples were collected and the total protein was extracted using extraction buffer (150 mM NaCl, 2.5 mM Tris-HCl, 1mM ethylenediaminetetraacetic acid, 10% glycerol, 0.1% NP40, 2% PVPP, 10 mM DTT, and 1× protease inhibitor cocktail). The sample was centrifuged at 12,000 rpm and 4 °C for 10 min, and the solubilized proteins were incubated with anti-Myc agarose beads for 3 h. The beads were washed three times with an extraction buffer containing 0.1% NP-40 and eluted with a 5× SDS sample buffer. The Western blot analysis analyzed the samples.

### 4.5. Yeast Two-Hybrid (Y2H) and Bimolecular Complementation Fluorescence (BiFC) Assays

Further, to analyze the interactions of CIPK31 with other proteins, a Match Maker yeast two-hybrid system (Y2H; Takara, Dalian, China) was used. The coding sequence of CIPK31 was subcloned into the pGBT9 (DNA-binding domain, BD) vector, and the coding sequences of the SLR1 and VOZ2 were subcloned into pGAD424 (activation domain, AD). The recombinant AD and BD plasmid pairs were co-transformed into the yeast strain Y2HGold, following the yeast transformation protocol (Takara, Dalian, China). Primers used for Y2H were listed in the supplemental Table S1.

The target sequences were cloned into the fluorescent protein vectors pXNGW and pXCGW, and these constructs were cotransformed into tobacco leaves using Agrobacterium-mediated transformation (GV3101) [51]. H2B-RFP was used as a nuclear marker, 36–48 h after transformation, the fluorescence signals were imaged using an Olympus FV3000 lasers scanning confocal microscope (Olympus, Tokyo, Japan), and 488 and 594 nm lasers were used to excite the fluorophores. 

### 4.6. Statistical Analysis

All statistical analyses were performed using the IBM SPSS statistics software (Version 20.0; IBM, Armonk, NY, USA); Student’s *t*-test was used to compare and determine statistically significant differences between the two groups. A one-way analysis of variance (ANOVA) was used to compare more than two groups. Different letters designate significantly different means by 2-way ANOVA + Duncan’s post hoc test (*p* < 0.05). All experiments were replicated in the laboratory at least three times.

## Data Availability

Data are contained within the article.

## Supplementary Materials

The following supporting information can be downloaded at: https://www.mdpi.com/article/10.3390/plants13101306/s1, Table S1: Primers used in this study.
